# Supplementary material for: Comparison of Oral Microbial Composition and Determinants Encoding Antimicrobial Resistance in Dogs and Their Owners
Source: Antibiotics (Basel). 2023 Oct 20;12(10):1554. doi: 10.3390/antibiotics12101554 (PMC10604839; doi:10.3390/antibiotics12101554)
Supplement: Supplementary file 1 [file antibiotics-12-01554-s001.zip › antibiotics-2589687-supplementary.pdf]

Table S1. **Bacterial species detected in oral cavity of dogs and their owners**

Legend	Taxonomy	Humans (H) %	Dogs (HA) %
	d__Archaea;p__Methanobacteriota;o__Methanobacteriales;f__Methanobacteriaceae;g__Methanobrevibacter_A;s__Methanobrevibacter_A oralis	0.0%	0.4%
	d__Bacteria;p__Actinobacteriota;o__Actinomycetales;f__Actinomycetaceae;g__Actinomyces;s__Actinomyces bowdenii	0.0%	0.4%
	d__Bacteria;p__Actinobacteriota;o__Actinomycetales;f__Actinomycetaceae;g__Actinomyces;s__Actinomyces bowdenii_B	0.0%	0.1%
	d__Bacteria;p__Actinobacteriota;o__Actinomycetales;f__Actinomycetaceae;g__Actinomyces;s__Actinomyces dentalis	0.2%	0.0%
	d__Bacteria;p__Actinobacteriota;o__Actinomycetales;f__Actinomycetaceae;g__Actinomyces;s__Actinomyces gerencseriae	0.7%	0.0%
	d__Bacteria;p__Actinobacteriota;o__Actinomycetales;f__Actinomycetaceae;g__Actinomyces;s__Actinomyces israelii	0.1%	0.0%
	d__Bacteria;p__Actinobacteriota;o__Actinomycetales;f__Actinomycetaceae;g__Actinomyces;s__Actinomyces johnsonii	0.3%	0.0%
	d__Bacteria;p__Actinobacteriota;o__Actinomycetales;f__Actinomycetaceae;g__Actinomyces;s__Actinomyces massiliensis	4.0%	0.0%
	d__Bacteria;p__Actinobacteriota;o__Actinomycetales;f__Actinomycetaceae;g__Actinomyces;s__Actinomyces naeslundii	2.2%	0.0%
	d__Bacteria;p__Actinobacteriota;o__Actinomycetales;f__Actinomycetaceae;g__Actinomyces;s__Actinomyces oris	9.8%	0.0%
	d__Bacteria;p__Actinobacteriota;o__Actinomycetales;f__Actinomycetaceae;g__Actinomyces;s__Actinomyces oris_A	2.1%	0.0%
	d__Bacteria;p__Actinobacteriota;o__Actinomycetales;f__Actinomycetaceae;g__Actinomyces;s__Actinomyces oris_C	0.1%	0.0%
	d__Bacteria;p__Actinobacteriota;o__Actinomycetales;f__Actinomycetaceae;g__Actinomyces;s__Actinomyces oris_D	0.0%	0.0%
	d__Bacteria;p__Actinobacteriota;o__Actinomycetales;f__Actinomycetaceae;g__Actinomyces;s__Actinomyces oris_E	0.5%	0.0%
	d__Bacteria;p__Actinobacteriota;o__Actinomycetales;f__Actinomycetaceae;g__Actinomyces;s__Actinomyces sp000195595	0.8%	0.0%
	d__Bacteria;p__Actinobacteriota;o__Actinomycetales;f__Actinomycetaceae;g__Actinomyces;s__Actinomyces sp000220835	7.1%	0.0%
	d__Bacteria;p__Actinobacteriota;o__Actinomycetales;f__Actinomycetaceae;g__Actinomyces;s__Actinomyces sp001278845	0.0%	0.0%
	d__Bacteria;p__Actinobacteriota;o__Actinomycetales;f__Actinomycetaceae;g__Actinomyces;s__Actinomyces sp002999235	0.2%	0.0%
	d__Bacteria;p__Actinobacteriota;o__Actinomycetales;f__Actinomycetaceae;g__Actinomyces;s__Actinomyces sp900323545	0.0%	0.0%
	d__Bacteria;p__Actinobacteriota;o__Actinomycetales;f__Actinomycetaceae;g__Actinomyces;s__Actinomyces weissii	0.0%	1.1%
	d__Bacteria;p__Actinobacteriota;o__Actinomycetales;f__Actinomycetaceae;g__Buchananella;s__Buchananella hordeovulneris	0.0%	0.9%
	d__Bacteria;p__Actinobacteriota;o__Actinomycetales;f__Actinomycetaceae;g__Pauljensenia;s__Pauljensenia canis	0.0%	1.9%
	d__Bacteria;p__Actinobacteriota;o__Actinomycetales;f__Actinomycetaceae;g__Pauljensenia;s__Pauljensenia hongkongensis	0.6%	0.0%
	d__Bacteria;p__Actinobacteriota;o__Actinomycetales;f__Actinomycetaceae;g__Pauljensenia;s__Pauljensenia meyeri	0.0%	0.0%
	d__Bacteria;p__Actinobacteriota;o__Actinomycetales;f__Actinomycetaceae;g__Pauljensenia;s__Pauljensenia odontolytica_A	4.3%	0.0%
	d__Bacteria;p__Actinobacteriota;o__Actinomycetales;f__Actinomycetaceae;g__Pauljensenia;s__Pauljensenia sp000185285	0.1%	0.0%
	d__Bacteria;p__Actinobacteriota;o__Actinomycetales;f__Actinomycetaceae;g__Pauljensenia;s__Pauljensenia sp000278725	0.0%	0.0%
	d__Bacteria;p__Actinobacteriota;o__Actinomycetales;f__Actinomycetaceae;g__Pauljensenia;s__Pauljensenia sp903645355	0.0%	0.2%
	d__Bacteria;p__Actinobacteriota;o__Actinomycetales;f__Actinomycetaceae;g__Peptidiphaga;s__Peptidiphaga gingivicola	0.1%	0.0%
	d__Bacteria;p__Actinobacteriota;o__Actinomycetales;f__Actinomycetaceae;g__Peptidiphaga;s__Peptidiphaga sp000466165	2.8%	0.0%
	d__Bacteria;p__Actinobacteriota;o__Actinomycetales;f__Microbacteriaceae;g__Canibacter;s__Canibacter oris	0.0%	0.0%
	d__Bacteria;p__Actinobacteriota;o__Actinomycetales;f__Microbacteriaceae;g__Canibacter;s__Canibacter sp003859945	0.0%	1.0%
	d__Bacteria;p__Actinobacteriota;o__Actinomycetales;f__Micrococcaceae;g__Rothia;s__Rothia aeria	1.2%	0.0%
	d__Bacteria;p__Actinobacteriota;o__Actinomycetales;f__Micrococcaceae;g__Rothia;s__Rothia dentocariosa	2.3%	0.0%
	d__Bacteria;p__Actinobacteriota;o__Actinomycetales;f__Micrococcaceae;g__Rothia;s__Rothia mucilaginosa	0.5%	0.0%
	d__Bacteria;p__Actinobacteriota;o__Actinomycetales;f__Micrococcaceae;g__Rothia;s__Rothia sp001808955	0.0%	0.0%

	d__Bacteria;p__Actinobacteriota;o__Coriobacteriales;f__Atopobiaceae;g__Olsenella_F;s__Olsenella_F sp001189515	0.1%	0.0%
	d__Bacteria;p__Actinobacteriota;o__Coriobacteriales;f__Atopobiaceae;g__RUG721;s__RUG721 sp004010535	0.0%	0.1%
	d__Bacteria;p__Actinobacteriota;o__Mycobacteriales;f__Mycobacteriaceae;g__Corynebacterium;s__Corynebacterium canis	0.0%	11.4%
	d__Bacteria;p__Actinobacteriota;o__Mycobacteriales;f__Mycobacteriaceae;g__Corynebacterium;s__Corynebacterium durum	0.2%	0.0%
	d__Bacteria;p__Actinobacteriota;o__Mycobacteriales;f__Mycobacteriaceae;g__Corynebacterium;s__Corynebacterium freiburgense	0.0%	2.9%
	d__Bacteria;p__Actinobacteriota;o__Mycobacteriales;f__Mycobacteriaceae;g__Corynebacterium;s__Corynebacterium matruchotii	1.2%	0.0%
	d__Bacteria;p__Actinobacteriota;o__Mycobacteriales;f__Mycobacteriaceae;g__Corynebacterium;s__Corynebacterium mustelae	0.0%	0.6%
	d__Bacteria;p__Actinobacteriota;o__Propionibacteriales;f__Propionibacteriaceae;g__Arachnia;s__Arachnia propionica	0.3%	0.0%
	d__Bacteria;p__Actinobacteriota;o__Propionibacteriales;f__Propionibacteriaceae;g__Arachnia;s__Arachnia propionica_B	0.0%	1.1%
	d__Bacteria;p__Actinobacteriota;o__Propionibacteriales;f__Propionibacteriaceae;g__Arachnia;s__Arachnia propionica_C	0.0%	0.4%
	d__Bacteria;p__Actinobacteriota;o__Propionibacteriales;f__Propionibacteriaceae;g__Arachnia;s__Arachnia rubra	0.6%	0.0%
	d__Bacteria;p__Actinobacteriota;o__Propionibacteriales;f__Propionibacteriaceae;g__Arachnia;s__Arachnia sp003932855	0.0%	2.5%
	d__Bacteria;p__Actinobacteriota;o__Propionibacteriales;f__Propionibacteriaceae;g__Arachnia;s__Arachnia sp900607225	0.1%	0.0%
	d__Bacteria;p__Actinobacteriota;o__Propionibacteriales;f__Propionibacteriaceae;g__Arachnia;s__Arachnia sp905372155	0.0%	0.0%
	d__Bacteria;p__Bacteroidota;o__Bacteroidales;f__Bacteroidaceae;g__Alloprevotella;s__Alloprevotella sp003859795	0.0%	0.4%
	d__Bacteria;p__Bacteroidota;o__Bacteroidales;f__Bacteroidaceae;g__Alloprevotella;s__Alloprevotella sp015259235	0.5%	0.0%
	d__Bacteria;p__Bacteroidota;o__Bacteroidales;f__Bacteroidaceae;g__Alloprevotella;s__Alloprevotella sp900095835	0.1%	0.0%
	d__Bacteria;p__Bacteroidota;o__Bacteroidales;f__Bacteroidaceae;g__Alloprevotella;s__Alloprevotella sp905371275	0.0%	0.0%
	d__Bacteria;p__Bacteroidota;o__Bacteroidales;f__Bacteroidaceae;g__Bacteroides;s__Bacteroides heparinolyticus	0.0%	0.1%
	d__Bacteria;p__Bacteroidota;o__Bacteroidales;f__Bacteroidaceae;g__Prevotella;s__Prevotella conceptionensis	0.1%	0.0%
	d__Bacteria;p__Bacteroidota;o__Bacteroidales;f__Bacteroidaceae;g__Prevotella;s__Prevotella denticola	0.0%	0.0%
	d__Bacteria;p__Bacteroidota;o__Bacteroidales;f__Bacteroidaceae;g__Prevotella;s__Prevotella loescheii	0.0%	0.0%
	d__Bacteria;p__Bacteroidota;o__Bacteroidales;f__Bacteroidaceae;g__Prevotella;s__Prevotella melaninogenica	0.3%	0.0%
	d__Bacteria;p__Bacteroidota;o__Bacteroidales;f__Bacteroidaceae;g__Prevotella;s__Prevotella melaninogenica_B	0.1%	0.0%
	d__Bacteria;p__Bacteroidota;o__Bacteroidales;f__Bacteroidaceae;g__Prevotella;s__Prevotella melaninogenica_C	0.0%	0.0%
	d__Bacteria;p__Bacteroidota;o__Bacteroidales;f__Bacteroidaceae;g__Prevotella;s__Prevotella nigrescens	0.4%	0.0%
	d__Bacteria;p__Bacteroidota;o__Bacteroidales;f__Bacteroidaceae;g__Prevotella;s__Prevotella oris	0.0%	0.0%
	d__Bacteria;p__Bacteroidota;o__Bacteroidales;f__Bacteroidaceae;g__Prevotella;s__Prevotella oulorum	0.1%	0.0%
	d__Bacteria;p__Bacteroidota;o__Bacteroidales;f__Bacteroidaceae;g__Prevotella;s__Prevotella salivae	0.0%	0.0%
	d__Bacteria;p__Bacteroidota;o__Bacteroidales;f__Bacteroidaceae;g__Prevotella;s__Prevotella sp000163055	0.0%	0.0%
	d__Bacteria;p__Bacteroidota;o__Bacteroidales;f__Bacteroidaceae;g__Prevotella;s__Prevotella sp000257925	0.0%	0.0%
	d__Bacteria;p__Bacteroidota;o__Bacteroidales;f__Bacteroidaceae;g__Prevotella;s__Prevotella sp000467895	0.1%	0.0%
	d__Bacteria;p__Bacteroidota;o__Bacteroidales;f__Bacteroidaceae;g__Prevotella;s__Prevotella sp003932845	0.0%	0.2%
	d__Bacteria;p__Bacteroidota;o__Bacteroidales;f__Bacteroidaceae;g__Prevotella;s__Prevotella veroralis	0.1%	0.0%
	d__Bacteria;p__Bacteroidota;o__Bacteroidales;f__Paludibacteraceae;g__F0058;s__F0058 sp000768855	0.0%	1.2%
	d__Bacteria;p__Bacteroidota;o__Bacteroidales;f__Paludibacteraceae;g__H1;s__H1 sp001653155	0.0%	0.6%
	d__Bacteria;p__Bacteroidota;o__Bacteroidales;f__Porphyromonadaceae;g__Porphyromonas;s__Porphyromonas catoniae	0.1%	0.0%
	d__Bacteria;p__Bacteroidota;o__Bacteroidales;f__Porphyromonadaceae;g__Porphyromonas;s__Porphyromonas crevioricanis	0.0%	0.6%
	d__Bacteria;p__Bacteroidota;o__Bacteroidales;f__Porphyromonadaceae;g__Porphyromonas;s__Porphyromonas gingivalis	0.0%	0.2%

	d__Bacteria;p__Bacteroidota;o__Bacteroidales;f__Porphyromonadaceae;g__Porphyromonas;s__Porphyromonas gingivicanis	0.0%	3.6%
	d__Bacteria;p__Bacteroidota;o__Bacteroidales;f__Porphyromonadaceae;g__Porphyromonas;s__Porphyromonas gulae	0.0%	14.6%
	d__Bacteria;p__Bacteroidota;o__Bacteroidales;f__Porphyromonadaceae;g__Porphyromonas;s__Porphyromonas macacae	0.0%	0.5%
	d__Bacteria;p__Bacteroidota;o__Bacteroidales;f__Porphyromonadaceae;g__Porphyromonas;s__Porphyromonas pasteri	0.4%	0.0%
	d__Bacteria;p__Bacteroidota;o__Bacteroidales;f__Porphyromonadaceae;g__Porphyromonas;s__Porphyromonas sp000467855	0.0%	0.0%
	d__Bacteria;p__Bacteroidota;o__Bacteroidales;f__Porphyromonadaceae;g__Porphyromonas;s__Porphyromonas sp000768875	0.0%	0.6%
	d__Bacteria;p__Bacteroidota;o__Bacteroidales;f__Porphyromonadaceae;g__Porphyromonas;s__Porphyromonas sp000769075	0.0%	1.7%
	d__Bacteria;p__Bacteroidota;o__Bacteroidales;f__Porphyromonadaceae;g__Porphyromonas_A;s__Porphyromonas_A cangingivalis	0.0%	8.1%
	d__Bacteria;p__Bacteroidota;o__Bacteroidales;f__Porphyromonadaceae;g__Porphyromonas_A;s__Porphyromonas_A canoris	0.0%	5.6%
	d__Bacteria;p__Bacteroidota;o__Bacteroidales;f__Tannerellaceae;g__Tannerella;s__Tannerella forsythia	0.0%	0.1%
	d__Bacteria;p__Bacteroidota;o__Bacteroidales;f__Tannerellaceae;g__Tannerella;s__Tannerella forsythia_A	0.0%	4.7%
	d__Bacteria;p__Bacteroidota;o__Bacteroidales;f__Tannerellaceae;g__Tannerella;s__Tannerella sp003033925	0.3%	0.0%
	d__Bacteria;p__Bacteroidota;o__Flavobacteriales;f__Flavobacteriaceae;g__Capnocytophaga;s__Capnocytophaga canimorsus	0.0%	0.5%
	d__Bacteria;p__Bacteroidota;o__Flavobacteriales;f__Flavobacteriaceae;g__Capnocytophaga;s__Capnocytophaga canis	0.0%	1.4%
	d__Bacteria;p__Bacteroidota;o__Flavobacteriales;f__Flavobacteriaceae;g__Capnocytophaga;s__Capnocytophaga cynodegmi	0.0%	1.5%
	d__Bacteria;p__Bacteroidota;o__Flavobacteriales;f__Flavobacteriaceae;g__Capnocytophaga;s__Capnocytophaga endodontalis	0.1%	0.0%
	d__Bacteria;p__Bacteroidota;o__Flavobacteriales;f__Flavobacteriaceae;g__Capnocytophaga;s__Capnocytophaga gingivalis	0.1%	0.0%
	d__Bacteria;p__Bacteroidota;o__Flavobacteriales;f__Flavobacteriaceae;g__Capnocytophaga;s__Capnocytophaga granulosa	0.0%	0.0%
	d__Bacteria;p__Bacteroidota;o__Flavobacteriales;f__Flavobacteriaceae;g__Capnocytophaga;s__Capnocytophaga leadbetteri	0.4%	0.0%
	d__Bacteria;p__Bacteroidota;o__Flavobacteriales;f__Flavobacteriaceae;g__Capnocytophaga;s__Capnocytophaga ochracea	0.1%	0.0%
	d__Bacteria;p__Bacteroidota;o__Flavobacteriales;f__Flavobacteriaceae;g__Capnocytophaga;s__Capnocytophaga sp905372595	0.0%	0.0%
	d__Bacteria;p__Bacteroidota;o__Flavobacteriales;f__Flavobacteriaceae;g__Capnocytophaga;s__Capnocytophaga sputigena	0.5%	0.0%
	d__Bacteria;p__Bacteroidota;o__Flavobacteriales;f__Weeksellaceae;g__Bergeyella;s__Bergeyella zoohelcum	0.0%	1.7%
	d__Bacteria;p__Bacteroidota;o__Flavobacteriales;f__Weeksellaceae;g__JABCPE02;s__JABCPE02 sp013333255	0.1%	0.0%
	d__Bacteria;p__Bacteroidota;o__Flavobacteriales;f__Weeksellaceae;g__JABCPE02;s__JABCPE02 sp013333875	0.1%	0.0%
	d__Bacteria;p__Campylobacterota;o__Campylobacterales;f__Campylobacteraceae;g__Campylobacter_A;s__Campylobacter_A concisus_AC	0.0%	0.0%
	d__Bacteria;p__Campylobacterota;o__Campylobacterales;f__Campylobacteraceae;g__Campylobacter_A;s__Campylobacter_A concisus_B	0.0%	0.0%
	d__Bacteria;p__Campylobacterota;o__Campylobacterales;f__Campylobacteraceae;g__Campylobacter_A;s__Campylobacter_A curvus	0.0%	0.0%
	d__Bacteria;p__Campylobacterota;o__Campylobacterales;f__Campylobacteraceae;g__Campylobacter_A;s__Campylobacter_A sp004803855	0.0%	0.1%
	d__Bacteria;p__Campylobacterota;o__Campylobacterales;f__Campylobacteraceae;g__Campylobacter_A;s__Campylobacter_A sp012978815	0.0%	0.9%
	d__Bacteria;p__Campylobacterota;o__Campylobacterales;f__Campylobacteraceae;g__Campylobacter_A;s__Campylobacter_A sp013201975	0.0%	0.8%
	d__Bacteria;p__Desulfobacterota;o__Desulfobulbales;f__Desulfobulbaceae;g__Desulfobulbus;s__Desulfobulbus oralis	0.0%	0.1%
	d__Bacteria;p__Desulfobacterota_I;o__Desulfovibrionales;f__Desulfomicrobiaceae;g__Desulfomicrobium;s__Desulfomicrobium orale	0.0%	1.4%
	d__Bacteria;p__Desulfobacterota_I;o__Desulfovibrionales;f__Desulfovibrionaceae;g__Desulfovibrio;s__Desulfovibrio sp003860215	0.0%	0.8%
	d__Bacteria;p__Firmicutes;o__Erysipelotrichales;f__Erysipelotrichaceae;g__Bulleidia;s__Bulleidia moorei	0.0%	0.0%
	d__Bacteria;p__Firmicutes;o__Erysipelotrichales;f__Erysipelotrichaceae;g__RQZE01;s__RQZE01 sp003858585	0.0%	0.9%
	d__Bacteria;p__Firmicutes;o__Lactobacillales;f__Aerococcaceae;g__Abiotrophia;s__Abiotrophia defectiva	0.0%	0.0%
	d__Bacteria;p__Firmicutes;o__Lactobacillales;f__Aerococcaceae;g__Granulicatella;s__Granulicatella adiacens	1.3%	0.0%
	d__Bacteria;p__Firmicutes;o__Lactobacillales;f__Aerococcaceae;g__Granulicatella;s__Granulicatella elegans	0.1%	0.0%

	d__Bacteria;p__Firmicutes;o__Lactobacillales;f__Streptococcaceae;g__Streptococcus;s__Streptococcus anginosus_C	0.0%	0.0%
	d__Bacteria;p__Firmicutes;o__Lactobacillales;f__Streptococcaceae;g__Streptococcus;s__Streptococcus australis	0.0%	0.0%
	d__Bacteria;p__Firmicutes;o__Lactobacillales;f__Streptococcaceae;g__Streptococcus;s__Streptococcus chosunense	0.1%	0.0%
	d__Bacteria;p__Firmicutes;o__Lactobacillales;f__Streptococcaceae;g__Streptococcus;s__Streptococcus constellatus	0.0%	0.0%
	d__Bacteria;p__Firmicutes;o__Lactobacillales;f__Streptococcaceae;g__Streptococcus;s__Streptococcus cristatus	0.4%	0.0%
	d__Bacteria;p__Firmicutes;o__Lactobacillales;f__Streptococcaceae;g__Streptococcus;s__Streptococcus cristatus_C	0.1%	0.0%
	d__Bacteria;p__Firmicutes;o__Lactobacillales;f__Streptococcaceae;g__Streptococcus;s__Streptococcus cristatus_E	0.0%	0.0%
	d__Bacteria;p__Firmicutes;o__Lactobacillales;f__Streptococcaceae;g__Streptococcus;s__Streptococcus dentisani	0.1%	0.0%
	d__Bacteria;p__Firmicutes;o__Lactobacillales;f__Streptococcaceae;g__Streptococcus;s__Streptococcus gordonii	0.6%	0.0%
	d__Bacteria;p__Firmicutes;o__Lactobacillales;f__Streptococcaceae;g__Streptococcus;s__Streptococcus halitosis	0.0%	0.0%
	d__Bacteria;p__Firmicutes;o__Lactobacillales;f__Streptococcaceae;g__Streptococcus;s__Streptococcus infantis	0.3%	0.0%
	d__Bacteria;p__Firmicutes;o__Lactobacillales;f__Streptococcaceae;g__Streptococcus;s__Streptococcus infantis_G	0.0%	0.0%
	d__Bacteria;p__Firmicutes;o__Lactobacillales;f__Streptococcaceae;g__Streptococcus;s__Streptococcus infantis_H	0.1%	0.0%
	d__Bacteria;p__Firmicutes;o__Lactobacillales;f__Streptococcaceae;g__Streptococcus;s__Streptococcus infantis_I	0.1%	0.0%
	d__Bacteria;p__Firmicutes;o__Lactobacillales;f__Streptococcaceae;g__Streptococcus;s__Streptococcus infantis_J	0.0%	0.0%
	d__Bacteria;p__Firmicutes;o__Lactobacillales;f__Streptococcaceae;g__Streptococcus;s__Streptococcus massiliensis	0.1%	0.0%
	d__Bacteria;p__Firmicutes;o__Lactobacillales;f__Streptococcaceae;g__Streptococcus;s__Streptococcus mitis	1.0%	0.0%
	d__Bacteria;p__Firmicutes;o__Lactobacillales;f__Streptococcaceae;g__Streptococcus;s__Streptococcus mitis_AC	2.2%	0.0%
	d__Bacteria;p__Firmicutes;o__Lactobacillales;f__Streptococcaceae;g__Streptococcus;s__Streptococcus mitis_AD	0.0%	0.0%
	d__Bacteria;p__Firmicutes;o__Lactobacillales;f__Streptococcaceae;g__Streptococcus;s__Streptococcus mitis_AF	0.1%	0.0%
	d__Bacteria;p__Firmicutes;o__Lactobacillales;f__Streptococcaceae;g__Streptococcus;s__Streptococcus mitis_AI	0.1%	0.0%
	d__Bacteria;p__Firmicutes;o__Lactobacillales;f__Streptococcaceae;g__Streptococcus;s__Streptococcus mitis_AK	0.1%	0.0%
	d__Bacteria;p__Firmicutes;o__Lactobacillales;f__Streptococcaceae;g__Streptococcus;s__Streptococcus mitis_AP	0.1%	0.0%
	d__Bacteria;p__Firmicutes;o__Lactobacillales;f__Streptococcaceae;g__Streptococcus;s__Streptococcus mitis_AT	0.0%	0.0%
	d__Bacteria;p__Firmicutes;o__Lactobacillales;f__Streptococcaceae;g__Streptococcus;s__Streptococcus mitis_AV	0.1%	0.0%
	d__Bacteria;p__Firmicutes;o__Lactobacillales;f__Streptococcaceae;g__Streptococcus;s__Streptococcus mitis_AW	0.2%	0.0%
	d__Bacteria;p__Firmicutes;o__Lactobacillales;f__Streptococcaceae;g__Streptococcus;s__Streptococcus mitis_AY	0.0%	0.0%
	d__Bacteria;p__Firmicutes;o__Lactobacillales;f__Streptococcaceae;g__Streptococcus;s__Streptococcus mitis_BB	0.2%	0.0%
	d__Bacteria;p__Firmicutes;o__Lactobacillales;f__Streptococcaceae;g__Streptococcus;s__Streptococcus mitis_BK	0.1%	0.0%
	d__Bacteria;p__Firmicutes;o__Lactobacillales;f__Streptococcaceae;g__Streptococcus;s__Streptococcus mitis_BM	0.6%	0.0%
	d__Bacteria;p__Firmicutes;o__Lactobacillales;f__Streptococcaceae;g__Streptococcus;s__Streptococcus mitis_BN	0.1%	0.0%
	d__Bacteria;p__Firmicutes;o__Lactobacillales;f__Streptococcaceae;g__Streptococcus;s__Streptococcus mitis_BO	0.0%	0.0%
	d__Bacteria;p__Firmicutes;o__Lactobacillales;f__Streptococcaceae;g__Streptococcus;s__Streptococcus mitis_BR	0.0%	0.0%
	d__Bacteria;p__Firmicutes;o__Lactobacillales;f__Streptococcaceae;g__Streptococcus;s__Streptococcus mitis_I	0.0%	0.0%
	d__Bacteria;p__Firmicutes;o__Lactobacillales;f__Streptococcaceae;g__Streptococcus;s__Streptococcus mitis_M	0.0%	0.0%
	d__Bacteria;p__Firmicutes;o__Lactobacillales;f__Streptococcaceae;g__Streptococcus;s__Streptococcus mitis_N	0.2%	0.0%
	d__Bacteria;p__Firmicutes;o__Lactobacillales;f__Streptococcaceae;g__Streptococcus;s__Streptococcus mitis_O	0.0%	0.0%
	d__Bacteria;p__Firmicutes;o__Lactobacillales;f__Streptococcaceae;g__Streptococcus;s__Streptococcus mitis_Q	0.1%	0.0%
	d__Bacteria;p__Firmicutes;o__Lactobacillales;f__Streptococcaceae;g__Streptococcus;s__Streptococcus mitis_S	3.8%	0.0%

	d__Bacteria;p__Firmicutes;o__Lactobacillales;f__Streptococcaceae;g__Streptococcus;s__Streptococcus mitis_W	0.0%	0.0%
	d__Bacteria;p__Firmicutes;o__Lactobacillales;f__Streptococcaceae;g__Streptococcus;s__Streptococcus mitis_Y	0.2%	0.0%
	d__Bacteria;p__Firmicutes;o__Lactobacillales;f__Streptococcaceae;g__Streptococcus;s__Streptococcus mutans	0.7%	0.0%
	d__Bacteria;p__Firmicutes;o__Lactobacillales;f__Streptococcaceae;g__Streptococcus;s__Streptococcus oralis	6.3%	0.0%
	d__Bacteria;p__Firmicutes;o__Lactobacillales;f__Streptococcaceae;g__Streptococcus;s__Streptococcus oralis_AC	0.1%	0.0%
	d__Bacteria;p__Firmicutes;o__Lactobacillales;f__Streptococcaceae;g__Streptococcus;s__Streptococcus oralis_AD	0.0%	0.0%
	d__Bacteria;p__Firmicutes;o__Lactobacillales;f__Streptococcaceae;g__Streptococcus;s__Streptococcus oralis_BA	0.3%	0.0%
	d__Bacteria;p__Firmicutes;o__Lactobacillales;f__Streptococcaceae;g__Streptococcus;s__Streptococcus oralis_BC	0.4%	0.0%
	d__Bacteria;p__Firmicutes;o__Lactobacillales;f__Streptococcaceae;g__Streptococcus;s__Streptococcus oralis_BD	0.1%	0.0%
	d__Bacteria;p__Firmicutes;o__Lactobacillales;f__Streptococcaceae;g__Streptococcus;s__Streptococcus oralis_BL	0.1%	0.0%
	d__Bacteria;p__Firmicutes;o__Lactobacillales;f__Streptococcaceae;g__Streptococcus;s__Streptococcus oralis_BM	0.0%	0.0%
	d__Bacteria;p__Firmicutes;o__Lactobacillales;f__Streptococcaceae;g__Streptococcus;s__Streptococcus oralis_J	0.1%	0.0%
	d__Bacteria;p__Firmicutes;o__Lactobacillales;f__Streptococcaceae;g__Streptococcus;s__Streptococcus oralis_K	0.0%	0.0%
	d__Bacteria;p__Firmicutes;o__Lactobacillales;f__Streptococcaceae;g__Streptococcus;s__Streptococcus oralis_M	0.0%	0.0%
	d__Bacteria;p__Firmicutes;o__Lactobacillales;f__Streptococcaceae;g__Streptococcus;s__Streptococcus oralis_S	1.2%	0.0%
	d__Bacteria;p__Firmicutes;o__Lactobacillales;f__Streptococcaceae;g__Streptococcus;s__Streptococcus oralis_W	0.2%	0.0%
	d__Bacteria;p__Firmicutes;o__Lactobacillales;f__Streptococcaceae;g__Streptococcus;s__Streptococcus oralis_Y	0.1%	0.0%
	d__Bacteria;p__Firmicutes;o__Lactobacillales;f__Streptococcaceae;g__Streptococcus;s__Streptococcus oralis_Z	1.2%	0.0%
	d__Bacteria;p__Firmicutes;o__Lactobacillales;f__Streptococcaceae;g__Streptococcus;s__Streptococcus parasanguinis_B	0.0%	0.0%
	d__Bacteria;p__Firmicutes;o__Lactobacillales;f__Streptococcaceae;g__Streptococcus;s__Streptococcus parasanguinis_F	0.0%	0.0%
	d__Bacteria;p__Firmicutes;o__Lactobacillales;f__Streptococcaceae;g__Streptococcus;s__Streptococcus pseudopneumoniae_C	0.2%	0.0%
	d__Bacteria;p__Firmicutes;o__Lactobacillales;f__Streptococcaceae;g__Streptococcus;s__Streptococcus sanguinis	6.0%	0.0%
	d__Bacteria;p__Firmicutes;o__Lactobacillales;f__Streptococcaceae;g__Streptococcus;s__Streptococcus sanguinis_C	0.1%	0.0%
	d__Bacteria;p__Firmicutes;o__Lactobacillales;f__Streptococcaceae;g__Streptococcus;s__Streptococcus sanguinis_E	0.1%	0.0%
	d__Bacteria;p__Firmicutes;o__Lactobacillales;f__Streptococcaceae;g__Streptococcus;s__Streptococcus sanguinis_G	0.1%	0.0%
	d__Bacteria;p__Firmicutes;o__Lactobacillales;f__Streptococcaceae;g__Streptococcus;s__Streptococcus sanguinis_H	1.0%	0.0%
	d__Bacteria;p__Firmicutes;o__Lactobacillales;f__Streptococcaceae;g__Streptococcus;s__Streptococcus shenyangsis	0.3%	0.0%
	d__Bacteria;p__Firmicutes;o__Lactobacillales;f__Streptococcaceae;g__Streptococcus;s__Streptococcus sp001556435	0.1%	0.0%
	d__Bacteria;p__Firmicutes;o__Lactobacillales;f__Streptococcaceae;g__Streptococcus;s__Streptococcus sp001808705	0.0%	0.0%
	d__Bacteria;p__Firmicutes;o__Lactobacillales;f__Streptococcaceae;g__Streptococcus;s__Streptococcus sp004166885	0.0%	0.0%
	d__Bacteria;p__Firmicutes;o__Lactobacillales;f__Streptococcaceae;g__Streptococcus;s__Streptococcus sp013394695	0.0%	0.0%
	d__Bacteria;p__Firmicutes;o__Lactobacillales;f__Streptococcaceae;g__Streptococcus;s__Streptococcus sp900104285	0.1%	0.0%
	d__Bacteria;p__Firmicutes;o__Lactobacillales;f__Streptococcaceae;g__Streptococcus;s__Streptococcus sp900555155	0.1%	0.0%
	d__Bacteria;p__Firmicutes;o__Lactobacillales;f__Streptococcaceae;g__Streptococcus;s__Streptococcus sp900766505	0.2%	0.0%
	d__Bacteria;p__Firmicutes;o__Lactobacillales;f__Streptococcaceae;g__Streptococcus;s__Streptococcus sp905221085	0.0%	0.0%
	d__Bacteria;p__Firmicutes;o__Lactobacillales;f__Streptococcaceae;g__Streptococcus;s__Streptococcus sp905221115	0.0%	0.0%
	d__Bacteria;p__Firmicutes;o__Lactobacillales;f__Streptococcaceae;g__Streptococcus;s__Streptococcus sp905221215	0.0%	0.0%
	d__Bacteria;p__Firmicutes;o__Lactobacillales;f__Streptococcaceae;g__Streptococcus;s__Streptococcus sp905221355	0.0%	0.0%
	d__Bacteria;p__Firmicutes;o__Lactobacillales;f__Streptococcaceae;g__Streptococcus;s__Streptococcus symci	1.7%	0.0%

	d__Bacteria;p__Firmicutes;o__Lactobacillales;f__Streptococcaceae;g__Streptococcus;s__Streptococcus vestibularis	0.9%	0.0%
	d__Bacteria;p__Firmicutes;o__Lactobacillales;f__Streptococcaceae;g__Streptococcus;s__Streptococcus xiaochunlingii	0.2%	0.0%
	d__Bacteria;p__Firmicutes;o__Staphylococcales;f__Gemellaceae;g__Gemella;s__Gemella haemolysans	0.1%	0.0%
	d__Bacteria;p__Firmicutes;o__Staphylococcales;f__Gemellaceae;g__Gemella;s__Gemella haemolysans_A	0.1%	0.0%
	d__Bacteria;p__Firmicutes;o__Staphylococcales;f__Gemellaceae;g__Gemella;s__Gemella haemolysans_B	0.2%	0.0%
	d__Bacteria;p__Firmicutes;o__Staphylococcales;f__Gemellaceae;g__Gemella;s__Gemella haemolysans_C	0.1%	0.0%
	d__Bacteria;p__Firmicutes;o__Staphylococcales;f__Gemellaceae;g__Gemella;s__Gemella morbillorum	0.2%	0.0%
	d__Bacteria;p__Firmicutes;o__Staphylococcales;f__Gemellaceae;g__Gemella;s__Gemella sp002871655	0.6%	0.0%
	d__Bacteria;p__Firmicutes;o__Staphylococcales;f__Gemellaceae;g__Gemella;s__Gemella sp900766305	0.2%	0.0%
	d__Bacteria;p__Firmicutes_A;o__Lachnospirales;f__Lachnospiraceae;g__Johnsonella;s__Johnsonella sp900766185	0.1%	0.0%
	d__Bacteria;p__Firmicutes_A;o__Lachnospirales;f__Lachnospiraceae;g__Lachnoanaerobaculum;s__Lachnoanaerobaculum saburreum	0.1%	0.0%
	d__Bacteria;p__Firmicutes_A;o__Lachnospirales;f__Lachnospiraceae;g__Lachnoanaerobaculum;s__Lachnoanaerobaculum sp000287675	0.0%	0.0%
	d__Bacteria;p__Firmicutes_A;o__Lachnospirales;f__Lachnospiraceae;g__Lachnoanaerobaculum;s__Lachnoanaerobaculum sp000296385	0.0%	0.0%
	d__Bacteria;p__Firmicutes_A;o__Lachnospirales;f__Vallitaleaceae;g__W11650;s__W11650 sp003858485	0.0%	0.5%
	d__Bacteria;p__Firmicutes_A;o__Peptostreptococcales;f__Anaerovoracaceae;g__Eubacterium_M;s__Eubacterium_M brachy	0.0%	0.0%
	d__Bacteria;p__Firmicutes_A;o__Peptostreptococcales;f__Anaerovoracaceae;g__Mogibacterium;s__Mogibacterium diversum	0.0%	0.0%
	d__Bacteria;p__Firmicutes_A;o__Peptostreptococcales;f__Filifactoraceae;g__Filifactor;s__Filifactor alocis	0.0%	0.1%
	d__Bacteria;p__Firmicutes_A;o__Peptostreptococcales;f__Peptostreptococcaceae;g__Peptostreptococcus;s__Peptostreptococcus canis	0.0%	0.4%
	d__Bacteria;p__Firmicutes_A;o__Peptostreptococcales;f__Peptostreptococcaceae;g__Peptostreptococcus;s__Peptostreptococcus stomatis	0.0%	0.0%
	d__Bacteria;p__Firmicutes_A;o__TANB77;f__CAG-508;g__CAG-793;s__CAG-793 sp013333035	0.0%	0.0%
	d__Bacteria;p__Firmicutes_A;o__Tissierellales;f__Peptoniphilaceae;g__Parvimonas;s__Parvimonas micra	0.1%	0.0%
	d__Bacteria;p__Firmicutes_A;o__Tissierellales;f__Peptoniphilaceae;g__W5053;s__W5053 sp000467935	0.0%	0.2%
	d__Bacteria;p__Firmicutes_C;o__Selenomonadales;f__Selenomonadaceae;g__Centipeda;s__Centipeda artemidis	0.0%	0.0%
	d__Bacteria;p__Firmicutes_C;o__Selenomonadales;f__Selenomonadaceae;g__Centipeda;s__Centipeda noxia	0.0%	0.0%
	d__Bacteria;p__Firmicutes_C;o__Selenomonadales;f__Selenomonadaceae;g__Centipeda;s__Centipeda sp000468035	0.0%	0.0%
	d__Bacteria;p__Firmicutes_C;o__Selenomonadales;f__Selenomonadaceae;g__Centipeda;s__Centipeda sp905372865	0.0%	0.0%
	d__Bacteria;p__Firmicutes_C;o__Veillonellales;f__Dialisteraceae;g__Dialister;s__Dialister invisus	0.0%	0.0%
	d__Bacteria;p__Firmicutes_C;o__Veillonellales;f__Megasphaeraceae;g__Anaeroglobus;s__Anaeroglobus micronuciformis	0.0%	0.0%
	d__Bacteria;p__Firmicutes_C;o__Veillonellales;f__Veillonellaceae;g__F0422;s__F0422 sp001553345	0.4%	0.0%
	d__Bacteria;p__Firmicutes_C;o__Veillonellales;f__Veillonellaceae;g__F0422;s__F0422 sp003992315	0.0%	0.0%
	d__Bacteria;p__Firmicutes_C;o__Veillonellales;f__Veillonellaceae;g__F0422;s__F0422 sp003999875	0.1%	0.0%
	d__Bacteria;p__Firmicutes_C;o__Veillonellales;f__Veillonellaceae;g__F0422;s__F0422 sp900766245	0.0%	0.0%
	d__Bacteria;p__Firmicutes_C;o__Veillonellales;f__Veillonellaceae;g__Veillonella;s__Veillonella nakazawae	0.0%	0.0%
	d__Bacteria;p__Firmicutes_C;o__Veillonellales;f__Veillonellaceae;g__Veillonella;s__Veillonella parvula	0.4%	0.0%
	d__Bacteria;p__Firmicutes_C;o__Veillonellales;f__Veillonellaceae;g__Veillonella;s__Veillonella parvula_A	4.0%	0.0%
	d__Bacteria;p__Firmicutes_C;o__Veillonellales;f__Veillonellaceae;g__Veillonella;s__Veillonella rogosae	0.0%	0.0%
	d__Bacteria;p__Firmicutes_C;o__Veillonellales;f__Veillonellaceae;g__Veillonella;s__Veillonella sp900757715	0.0%	0.0%
	d__Bacteria;p__Fusobacteriota;o__Fusobacteriales;f__Fusobacteriaceae;g__Fusobacterium;s__Fusobacterium animalis	0.0%	0.0%
	d__Bacteria;p__Fusobacteriota;o__Fusobacteriales;f__Fusobacteriaceae;g__Fusobacterium;s__Fusobacterium canifelinum	0.0%	0.1%

	d__Bacteria;p__Fusobacteriota;o__Fusobacteriales;f__Fusobacteriaceae;g__Fusobacterium;s__Fusobacterium periodonticum_D	0.0%	0.0%
	d__Bacteria;p__Fusobacteriota;o__Fusobacteriales;f__Fusobacteriaceae;g__Fusobacterium;s__Fusobacterium polymorphum	1.0%	0.0%
	d__Bacteria;p__Fusobacteriota;o__Fusobacteriales;f__Fusobacteriaceae;g__Fusobacterium;s__Fusobacterium russii	0.0%	0.1%
	d__Bacteria;p__Fusobacteriota;o__Fusobacteriales;f__Leptotrichiaceae;g__Leptotrichia;s__Leptotrichia buccalis	0.0%	0.0%
	d__Bacteria;p__Fusobacteriota;o__Fusobacteriales;f__Leptotrichiaceae;g__Leptotrichia;s__Leptotrichia hofstadii	0.1%	0.0%
	d__Bacteria;p__Fusobacteriota;o__Fusobacteriales;f__Leptotrichiaceae;g__Leptotrichia;s__Leptotrichia hongkongensis	0.0%	0.0%
	d__Bacteria;p__Fusobacteriota;o__Fusobacteriales;f__Leptotrichiaceae;g__Leptotrichia;s__Leptotrichia massiliensis	0.0%	0.0%
	d__Bacteria;p__Fusobacteriota;o__Fusobacteriales;f__Leptotrichiaceae;g__Leptotrichia;s__Leptotrichia sp013394795	0.0%	0.0%
	d__Bacteria;p__Fusobacteriota;o__Fusobacteriales;f__Leptotrichiaceae;g__Leptotrichia;s__Leptotrichia wadei	0.3%	0.0%
	d__Bacteria;p__Fusobacteriota;o__Fusobacteriales;f__Leptotrichiaceae;g__Leptotrichia_A;s__Leptotrichia_A sp000469505	0.0%	0.0%
	d__Bacteria;p__Fusobacteriota;o__Fusobacteriales;f__Leptotrichiaceae;g__Leptotrichia_A;s__Leptotrichia_A sp001274535	0.1%	0.0%
	d__Bacteria;p__Fusobacteriota;o__Fusobacteriales;f__Leptotrichiaceae;g__Leptotrichia_A;s__Leptotrichia_A sp905371725	0.0%	0.0%
	d__Bacteria;p__Fusobacteriota;o__Fusobacteriales;f__Leptotrichiaceae;g__Pseudoleptotrichia;s__Pseudoleptotrichia sp003932895	0.0%	0.1%
	d__Bacteria;p__Patescibacteria;o__Saccharimonadales;f__Nanosynbacteraceae;g__Nanosynbacter;s__Nanosynbacter lyticus	0.0%	0.0%
	d__Bacteria;p__Patescibacteria;o__Saccharimonadales;f__Saccharimonadaceae;g__Saccharimonas;s__Saccharimonas sp010202265	0.0%	0.0%
	d__Bacteria;p__Patescibacteria;o__Saccharimonadales;f__Saccharimonadaceae;g__Saccharimonas;s__Saccharimonas sp013333625	0.0%	0.0%
	d__Bacteria;p__Patescibacteria;o__Saccharimonadales;f__Saccharimonadaceae;g__Saccharimonas;s__Saccharimonas sp013333645	0.0%	0.0%
	d__Bacteria;p__Patescibacteria;o__Saccharimonadales;f__Saccharimonadaceae;g__Saccharimonas;s__Saccharimonas sp013333795	0.0%	0.0%
	d__Bacteria;p__Patescibacteria;o__Saccharimonadales;f__Saccharimonadaceae;g__Saccharimonas;s__Saccharimonas sp018127705	0.1%	0.0%
	d__Bacteria;p__Patescibacteria;o__Saccharimonadales;f__Saccharimonadaceae;g__Saccharimonas;s__Saccharimonas sp905373835	0.0%	0.0%
	d__Bacteria;p__Patescibacteria;o__Saccharimonadales;f__UBA10027;g__SDRW01;s__SDRW01 sp007845485	0.0%	0.0%
	d__Bacteria;p__Proteobacteria;o__Burkholderiales;f__Burkholderiaceae;g__Lampropedia;s__Lampropedia sp002285265	0.0%	0.0%
	d__Bacteria;p__Proteobacteria;o__Burkholderiales;f__Burkholderiaceae;g__Lampropedia;s__Lampropedia sp002285285	0.0%	2.0%
	d__Bacteria;p__Proteobacteria;o__Burkholderiales;f__Burkholderiaceae;g__Lampropedia;s__Lampropedia sp003703475	0.0%	7.6%
	d__Bacteria;p__Proteobacteria;o__Burkholderiales;f__Burkholderiaceae;g__Lautropia;s__Lautropia dentalis	0.1%	0.0%
	d__Bacteria;p__Proteobacteria;o__Burkholderiales;f__Burkholderiaceae;g__Lautropia;s__Lautropia mirabilis	1.2%	0.0%
	d__Bacteria;p__Proteobacteria;o__Burkholderiales;f__Burkholderiaceae;g__Ottowia;s__Ottowia sp001262075	0.0%	0.0%
	d__Bacteria;p__Proteobacteria;o__Burkholderiales;f__Burkholderiaceae;g__Ottowia;s__Ottowia sp003859965	0.0%	0.1%
	d__Bacteria;p__Proteobacteria;o__Burkholderiales;f__Neisseriaceae;g__Conchiformibius;s__Conchiformibius steedae	0.0%	0.2%
	d__Bacteria;p__Proteobacteria;o__Burkholderiales;f__Neisseriaceae;g__Eikenella;s__Eikenella corrodens	0.0%	0.0%
	d__Bacteria;p__Proteobacteria;o__Burkholderiales;f__Neisseriaceae;g__Eikenella;s__Eikenella shayeganii	0.0%	0.3%
	d__Bacteria;p__Proteobacteria;o__Burkholderiales;f__Neisseriaceae;g__Kingella_B;s__Kingella_B oralis	0.1%	0.0%
	d__Bacteria;p__Proteobacteria;o__Burkholderiales;f__Neisseriaceae;g__Neisseria;s__Neisseria animaloris	0.0%	0.2%
	d__Bacteria;p__Proteobacteria;o__Burkholderiales;f__Neisseriaceae;g__Neisseria;s__Neisseria canis	0.0%	0.6%
	d__Bacteria;p__Proteobacteria;o__Burkholderiales;f__Neisseriaceae;g__Neisseria;s__Neisseria cinerea	0.2%	0.0%
	d__Bacteria;p__Proteobacteria;o__Burkholderiales;f__Neisseriaceae;g__Neisseria;s__Neisseria dumasiana	0.0%	1.2%
	d__Bacteria;p__Proteobacteria;o__Burkholderiales;f__Neisseriaceae;g__Neisseria;s__Neisseria elongata	0.5%	0.0%
	d__Bacteria;p__Proteobacteria;o__Burkholderiales;f__Neisseriaceae;g__Neisseria;s__Neisseria mucosa	0.1%	0.0%
	d__Bacteria;p__Proteobacteria;o__Burkholderiales;f__Neisseriaceae;g__Neisseria;s__Neisseria mucosa_A	3.1%	0.0%

	d__Bacteria;p__Proteobacteria;o__Burkholderiales;f__Neisseriaceae;g__Neisseria;s__Neisseria sicca	0.1%	0.0%
	d__Bacteria;p__Proteobacteria;o__Burkholderiales;f__Neisseriaceae;g__Neisseria;s__Neisseria sp000090875	0.2%	0.0%
	d__Bacteria;p__Proteobacteria;o__Burkholderiales;f__Neisseriaceae;g__Neisseria;s__Neisseria sp000186165	0.0%	0.0%
	d__Bacteria;p__Proteobacteria;o__Burkholderiales;f__Neisseriaceae;g__Neisseria;s__Neisseria subflava	0.0%	0.0%
	d__Bacteria;p__Proteobacteria;o__Burkholderiales;f__Neisseriaceae;g__Neisseria;s__Neisseria weaveri	0.0%	0.2%
	d__Bacteria;p__Proteobacteria;o__Burkholderiales;f__Neisseriaceae;g__Neisseria;s__Neisseria zoodegmatis	0.0%	0.4%
	d__Bacteria;p__Proteobacteria;o__Cardiobacteriales;f__Cardiobacteriaceae;g__Cardiobacterium;s__Cardiobacterium hominis	0.2%	0.0%
	d__Bacteria;p__Proteobacteria;o__Enterobacterales;f__Pasteurellaceae;g__Aggregatibacter;s__Aggregatibacter aphrophilus	0.0%	0.0%
	d__Bacteria;p__Proteobacteria;o__Enterobacterales;f__Pasteurellaceae;g__Aggregatibacter;s__Aggregatibacter segnis_A	0.0%	0.0%
	d__Bacteria;p__Proteobacteria;o__Enterobacterales;f__Pasteurellaceae;g__Aggregatibacter;s__Aggregatibacter sp000466335	0.1%	0.0%
	d__Bacteria;p__Proteobacteria;o__Enterobacterales;f__Pasteurellaceae;g__Frederiksenia;s__Frederiksenia canicola	0.0%	0.2%
	d__Bacteria;p__Proteobacteria;o__Enterobacterales;f__Pasteurellaceae;g__Haemophilus;s__Haemophilus haemolyticus	0.2%	0.0%
	d__Bacteria;p__Proteobacteria;o__Enterobacterales;f__Pasteurellaceae;g__Haemophilus;s__Haemophilus seminalis	0.3%	0.0%
	d__Bacteria;p__Proteobacteria;o__Enterobacterales;f__Pasteurellaceae;g__Haemophilus_D;s__Haemophilus_D parainfluenzae	1.6%	0.0%
	d__Bacteria;p__Proteobacteria;o__Enterobacterales;f__Pasteurellaceae;g__Haemophilus_D;s__Haemophilus_D parainfluenzae_M	1.3%	0.0%
	d__Bacteria;p__Proteobacteria;o__Enterobacterales;f__Pasteurellaceae;g__Haemophilus_D;s__Haemophilus_D parainfluenzae_N	0.2%	0.0%
	d__Bacteria;p__Proteobacteria;o__Enterobacterales;f__Pasteurellaceae;g__Haemophilus_D;s__Haemophilus_D parainfluenzae_O	0.0%	0.0%
	d__Bacteria;p__Proteobacteria;o__Enterobacterales;f__Pasteurellaceae;g__Haemophilus_D;s__Haemophilus_D sp001815355	0.0%	0.0%
	d__Bacteria;p__Proteobacteria;o__Enterobacterales;f__Pasteurellaceae;g__Haemophilus_D;s__Haemophilus_D sp900756155	0.0%	0.0%
	d__Bacteria;p__Proteobacteria;o__Enterobacterales;f__Pasteurellaceae;g__Haemophilus_D;s__Haemophilus_D sp905215245	0.0%	0.0%
	d__Bacteria;p__Proteobacteria;o__Enterobacterales;f__Pasteurellaceae;g__Pasteurella;s__Pasteurella canis	0.0%	0.5%
	d__Bacteria;p__Proteobacteria;o__Enterobacterales;f__Pasteurellaceae;g__Pasteurella;s__Pasteurella dagmatis	0.0%	0.1%
	d__Bacteria;p__Proteobacteria;o__Enterobacterales;f__Pasteurellaceae;g__Pasteurella;s__Pasteurella multocida_A	0.0%	0.2%
	d__Bacteria;p__Proteobacteria;o__Pseudomonadales;f__Moraxellaceae;g__Moraxella;s__Moraxella sp002224245	0.0%	1.7%
	d__Bacteria;p__Proteobacteria;o__Xanthomonadales;f__Xanthomonadaceae;g__Lysobacter_B;s__Lysobacter_B sp002798195	0.0%	0.2%
	d__Bacteria;p__Proteobacteria;o__Xanthomonadales;f__Xanthomonadaceae;g__Lysobacter_B;s__Lysobacter_B sp002798275	0.0%	0.1%
	d__Bacteria;p__Proteobacteria;o__Xanthomonadales;f__Xanthomonadaceae;g__Lysobacter_B;s__Lysobacter_B sp002798295	0.0%	0.4%
	d__Bacteria;p__Spirochaetota;o__Treponematales;f__Treponemataceae;g__Treponema;s__Treponema sp010365865	0.1%	0.0%
	d__Bacteria;p__Synergistota;o__Synergistales;f__Aminobacteriaceae;g__CAJPSE01;s__CAJPSE01 sp003860125	0.0%	4.4%
